# The limits and boundaries of digital disconnection

**DOI:** 10.1177/0163443720922054

**Published:** 2020-05-04

**Authors:** Emiliano Treré, Simone Natale, Emily Keightley, Aswin Punathambekar

**Affiliations:** Cardiff University, UK; Loughborough University, UK; University of Virginia, USA

**Keywords:** connectivity, digital, disconnection, global south, infrastructure, platforms

## Abstract

This editorial introduces a themed section aimed to spark further reflections on the limits and boundaries of disconnection as a form of critique, activism and response to the pervasiveness of digital devices, platforms, and infrastructures. We outline two key limits in current thinking about disconnection: first, the universalist discourse of disconnection, which contrasts with the reality of a profound inequality of access to both connection and disconnection across the globe, and second, the fact that connectivity not only involves digital media users but also those who are materially not connected to the network. This introduction also reflects on the changing meanings of being connected and disconnected to digital networks and platforms at a time when the Covid-19 pandemic forces many people around the world to remain physically separated from others due to lockdown and quarantine measures.

Connectivity has been a potent keyword over the past decade. The growing availability of fast, cheap Internet access via mobile made the constant connection to social networks and Internet-based resources an everyday experience for larger masses of people around the world. As part of this process, the right for universal connection was invoked by corporations and governments alike to articulate a new and decidedly optimistic vision of inclusion. Yet the apparently benign endeavor of ‘connecting people’ (as Facebook’s marketing strategy puts it, see Karppi, 2018) could only partially conceal the fact that users were provided with just the illusion of control over their own connectivity. It has become evident not only to media critics but also to many everyday users that constant connectivity comes at a steep price. The emotional and psychological cost of constant access to Internet-based devices has been at the center of growing concerns (Alter, 2017). The revelation of the close links between the political economy of surveillance and the business model of most powerful digital corporations also sparked criticism (Nieborg and Helmond, 2019). What was heralded as the era of universal connection was therefore accompanied by a complementary aspiration to disconnection: the ability to sever links between ourselves and the networked infrastructures of information societies.

The Covid-19 pandemic in which we are caught up in as we draft these lines has radically altered the delicate balance between digital connection and disconnection. This public health crisis has unexpectedly pushed most activities that were performed offline to the online world, generating an unprecedented acceleration and intensification of digitally enabled and online-only activities. In this scenario, it becomes even more relevant to explore how the sudden shift to hyper-connectivity is redefining our practices, impacting our wellbeing, and redrawing the limits and boundaries of digital disconnection. This global emergency is magnifying the many contradictions of our digital society. It has become clear that reliable Internet connection and access to computing devices is not a luxury but rather, a social need and a public utility. It is the backbone of our work and leisure activities and the infrastructure for the maintaining and nurturing of our interpersonal ties and affects. By the same token, as we push the time of daily life to its digitally connected limits, we discern that now more than ever, being able to disconnect is just as crucial. Faced with tremendous levels of stress and anxiety, we find ourselves needing to slow down and switch off from the online world. In these extreme circumstances, both the possibility to connect and the ability to disconnect manifest themselves as needs and possibilities that are ineluctably shaped by socio-economic and cultural forces and factors including status, class, nationality, gender, (dis)ability, and so on.

While disconnection promises a potential liberation or redemption from the contradictions of contemporary digital capitalism, it remains a problematic issue both at a conceptual and practical level. It is true that we live in times of ‘coerced digital participation’ (Barassi, 2019), yet it remains unclear to what extent disconnecting from digital networks can be an antidote to this problem. The essays collected in this Crosscurrents themed section contribute to ongoing reflections on the limits and boundaries of disconnection as a form of critique, activism and response to the power that digital media technologies and corporations held over users. Rather than presenting merely a critique to ongoing approaches and practices of digital disconnection, we have asked authors to put forward theoretical and pragmatic means that, we hope, will help spark new ideas and discussions on the topic.

On the whole, this themed section identifies two crucial limits in contemporary practices and concepts of disconnection. The first limit is what we might call the ‘universalism of disconnection’. Until now, disconnection has emerged mainly as a discourse that privileges certain populations and disregards others, supporting normalizing conceptions of what it means to be disconnected. There is a tendency to privilege educated actors, and a limited capacity to engage with diverse publics. This universalism significantly reduces the possible manifestations of disconnection to specific categories of white, educated people with high purchasing power in the West, thus disregarding the ways disconnection is performed and lived in other parts of the world and indeed, among vulnerable minorities within wealthy Western nations.

As with any aspect of media use and consumption, we must approach the pragmatics and politics of disconnection in a radically contextualized manner, taking into account the profoundly uneven ways in which people in varied places and times access the Internet. For millions of people across the Global South, disconnection is nothing akin to a matter of choice, one that can be indulged in every now and then. In countries and regions with poor communications infrastructure and low Internet penetration, large numbers of people are simply not able to connect, forming what Straumann and Graham (2016) have named an ‘Archipelago of Disconnection’. In other contexts, people contend with intermittent and precarious connections to online environments. Disconnection can also be forced, and at times is part of a violent politics of enclosure and occupation marked by intense state surveillance and control (Tawil-Souri, 2012). For instance, in August 2019, the right-wing BJP regime invoked sections of a British colonial-era law (Indian Telegraph Act of 1885) to suspend Internet services in the highly militarized north Indian state of Kashmir. At the time of this writing, Kashmiri citizens have lived through the longest Internet shutdown ever imposed in a democracy. We cannot overstate the importance of understanding digital disconnection when stable connectivity is *not* the default (Lim, 2020; Pype, 2019).

A second set of limitations has to do with the very character of connection in the digital age. In data-driven societies, connectivity is not just something that is offered or imposed on users, but something that aspires to encompass even those who are technically not connected to the network. Data are constantly produced not just by ourselves but by our friends, relatives and ‘contacts’. In the same moment as one disconnects from social networks or buys a low-key mobile phone with no Internet connection, their picture might appear in the pictures uploaded by other users or in the database of a governmental agency. One wonders, in this sense, if digital disconnection provides any solution to the problems we are facing, or if it needs to be at least reformulated in order to create real opportunities for shaping a better future in digital societies.

Taken together, the three articles that follow provide responses to these and other questions, reimagining both the meanings and the boundaries of digital disconnection. In the first article featured in this themed section, Taina Bucher (2020) asks if disconnection can be considered a viable form of agency in contemporary datafied societies. She explores how the logics of datafication and predictive analytics make it difficult to abstain from and opt out from digital platforms and services. In doing so, Bucher proposes a radical rethinking of the notion of individuality and collectivity in the digital age. In the second article, Merlyna Lim (2020) underlines the inequality of power and infrastructure that inform the meaning and the very possibility of enacting disconnection in the Global South. Building on her research in Indonesia, she puts forward the notion of ‘dis/connection’ as a new framework for disconnection studies to underline how the interplay between connection and disconnection serves as a tactic and a technique of both repression and resistance. Finally, in the third article, Simone Natale and Emiliano Treré (2020) point to the thin line between disconnection and disengagement to argue that disconnection may function as a trigger but also as an antidote to critique. In contrast with disconnection, they draw from a long tradition of critical thought, from Joseph Weizenbaum to Jaron Lanier passing through hacktivism, which demonstrates that engagement with digital technologies provides the necessary insight to advance critique and put forward alternative approaches to digital media. Through the concept of ‘Disconnection-through-Engagement’, Natale and Treré propose situated practices that mobilize disconnection in order to improve critical engagement with digital technologies and platforms, such as hybridity, anonymity, and hacking.

We regard this Crosscurrents themed issue as part of a broader conversation on global digital cultures that this journal has facilitated (Arora and Rangaswamy, 2013; Kuntsman and Miyake, 2019; Plantin and Punathambekar, 2019; Qiu, 2016; Sardá et al., 2019; Willems, 2019). We would like, therefore, to invite scholars from across the world to respond to these articles and add new questions and perspectives on the politics and pragmatics of digital disconnection, in line with Crosscurrents’ mandate to foster conversations and discussions in the field.
